# Protocol for optical clearing and imaging of fluorescently labeled *ex vivo* rat brain slices

**DOI:** 10.1016/j.xpro.2022.102041

**Published:** 2023-01-18

**Authors:** Jessica Giacomoni, Mette Habekost, Efrain Cepeda-Prado, Bengt Mattsson, Daniella Rylander Ottosson, Malin Parmar, Janko Kajtez

**Affiliations:** 1 Developmental and Regenerative Neurobiology, Wallenberg Neuroscience Center, Lund Stem Cell Center, Department of Experimental Medical Science, Lund University, Lund, Sweden; 2 Center for Neuroscience, University of Copenhagen Faculty of Health and Medical Sciences, Copenhagen, Denmark; 3 Regenerative Neurophysiology, Wallenberg Neuroscience Center, Lund Stem Cell Center, Department of Experimental Medical Science, Lund University, Lund, Sweden

**Keywords:** Cell Biology, Cell culture, Microscopy, Model Organisms, Neuroscience

## Abstract

Tissue clearing is commonly used for whole-brain imaging but seldom used for brain slices. Here, we present a simple protocol to slice, immunostain, and clear sections of adult rat brains for subsequent high-resolution confocal imaging. The protocol does not require toxic reagents or specialized equipment. We also provide instructions for culturing of rat brain slices free floating on permeable culture inserts, maintained in regular CO_2_ incubators, and handled only at media change.

## Highlights

• Preparation of acute brain slices from adult rats
• Description of organotypic brain slice culturing and immunostaining
• Tissue clearing through dehydration, lipid removal, and refractive index matching
• Confocal imaging of flattened brain slices

## Abstract (editor highlights)

Publisher’s note: Undertaking any experimental protocol requires adherence to local institutional guidelines for laboratory safety and ethics.

## Graphical abstract

**Figure:**
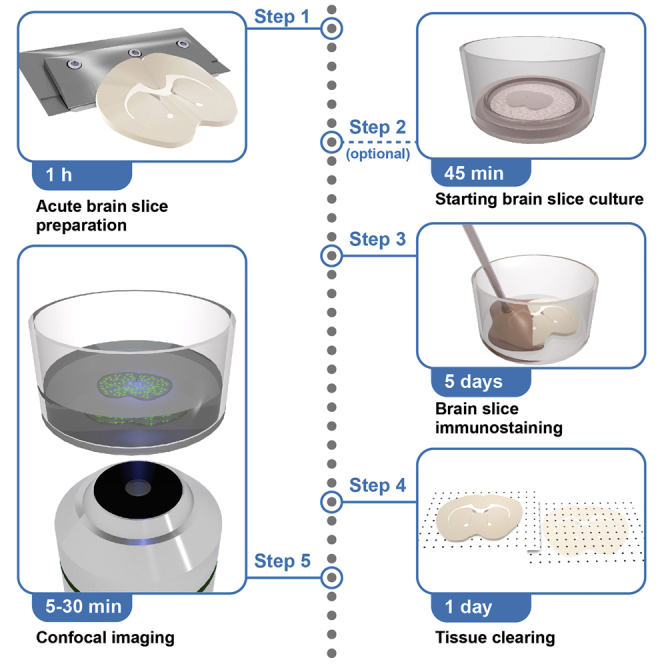

## Before you begin

Brain slices, either as acute preparations or as organotypic cultures, represent physiologically relevant three-dimensional *ex vivo* platform widely used for the investigation of neuronal circuitry and cellular/molecular mechanism of the brain. For decades, they have been invaluable for electrophysiological studies,^1^^,^^2^^,^^3^ modeling of neurodegenerative diseases,^4^^,^^5^ and evaluation of therapeutic options prior to *in vivo* investigations.^6^ However, microscopy strategies coupled with optical tissue clearing remain largely unexplored for brain slices even though deep tissue imaging with cellular resolution poses a major challenge in non-cleared tissue.^7^ Here, we describe a detailed protocol for cutting, immunofluorescent labeling, and clearing to facilitate imaging of acute/cultured brain slices. The methodology provides a low-cost and simple solution for optical access throughout the entire thickness of the brain slice (250–300 μm) to reveal structural information otherwise lost in non-cleared tissue. Refractive index matching is based on ethyl cinnamate, a non-toxic compound that can be handled and used on microscope systems without special safety measures.^8^^,^^9^ Furthermore, the tissue flattening demonstrated here reduces time for image acquisition and leads to smaller file sizes. As such, this simple and reliable protocol is intended to be routinely used for imaging and histological analysis of *ex vivo* brain slices.

Notably, the culture system presented here is suitable for *ex vivo* pre-clinical validation of therapeutics, viral transduction screening, and genetic manipulations (i.e., transgene delivery^10^). We have tested this protocol using *ex vivo* preparations from female Sprague-Dawley and athymic nude rats of four to fifteen months of age. Furthermore, it is compatible with rats that have been treated with compounds before brain collection such as unilateral 6-hydroxydopamine (6-OHDA) injection into the median forebrain bundle assessed by amphetamine-induced rotations and with rats that have received cell transplantation^11^ and injections of pseudotyped rabies vectors.^12^^,^^13^

### Institutional permissions

All procedures were performed in accordance with the European Union Directive (2010/63/EU) and approved by the local ethical committee at Lund University, as well as the Swedish Department of Agriculture (Jordbruksverket). Female Sprague-Dawley (SD) rats were purchased from Charles River and female Hsd:RH-Foxn1^rnu^ rats from Envigo. All rats were housed in ventilated cages with *ad libitum* access to food and water under a 12 h light/dark cycle. Neither the strain nor sex of the animal is important for this protocol.

## Key resources table

**Table:** 

REAGENT or RESOURCE	SOURCE	IDENTIFIER
**Antibodies**		

Donkey anti-rabbit IgG (H + L), Alexa Fluor 647 (1:200)	Jackson ImmunoResearch	Cat#711-605-152; RRID: AB_2492288
Rabbit polyclonal anti-Iba1 (1:1000)	FUJIFILM Wako	Cat#019-19741; RRID: AB_839504

**Chemicals, peptides, and recombinant proteins**		

1-Propanol	Sigma-Aldrich	279544
4-(2-Hydroxyethyl)-1-piperazineethanesulfonic acid, HEPES	Sigma-Aldrich	H3375-1KG
Antibiotic-Antimycotic	Thermo Fisher Scientific	15240-096
B-27 Supplement (minus vitamin A)	Thermo Fisher Scientific	12587-010
BrainPhys Neuronal Medium	STEMCELL Technologies	05790
Calcium chloride, CaCl_2_**·**2H_2_O	Sigma-Aldrich	C1016-500G
D-(+)-Glucose	Sigma-Aldrich	G8270-1KG
DAPI (1:1000)	Sigma-Aldrich	D9542
Dichloromethane, DCM	Acros Organics	AC124050010
Dipotassium phosphate, K_2_HPO_4_	Merck Millipore	1.05104.1000
Donkey serum	Biowest	S2170-500
Ethanol	Solveco	1054
Ethyl cinnamate	Sigma-Aldrich	112372
GlutaMAX Supplement (100X)	Thermo Fisher Scientific	35050038
Hanks' Balanced Salt Solution, HBSS (no calcium, no magnesium, no phenol red)	Thermo Fisher Scientific	14175-046
Hydrochloric acid, HCl	Sigma-Aldrich	258148-2.5L
L-Glutamine 200 mM	Thermo Fisher Scientific	25030-081
Methanol	Merck Millipore	1.06009.1000
Magnesium chloride hexahydrate, MgCl_2_·6H_2_O	Sigma-Aldrich	M2670-100G
Magnesium sulfate heptahydrate, MgSO_4_**·**7H_2_O	Sigma-Aldrich	230391-500G
N-Methyl-D-glucamine, NMDG	Sigma-Aldrich	M2004-500G
Penicillin-Streptomycin (10,000 U/mL)	Thermo Fisher Scientific	15140-122
Potassium chloride, KCl	Sigma-Aldrich	P3911-500G
Sodium ascorbate	Sigma-Aldrich	A7631-25G
Sodium azide, NaN_3_	Sigma-Aldrich	S2002
Sodium bicarbonate, NaHCO_3_	Sigma-Aldrich	S5761-500G
Sodium chloride, NaCl	Thermo Fisher Scientific	7647-14-5
Sodium phosphate monobasic, NaH_2_PO_4_	Sigma-Aldrich	S9638-25G
Sodium pyruvate	Sigma-Aldrich	P2256-25G
Thiourea	Sigma-Aldrich	T8656-50G
Triton X-100	MP Biomedicals	807426
Paraformaldehyde 4%, PFA	Sigma-Aldrich	30525-89-4
Pentobarbital sodium	APL Sweden	338327
Potassium dihydrogen phosphate, KH_2_PO_4_	J.T. Baker	15108936

**Experimental models: Organisms/strains**		

Rat: adult (4–15 months) female Sprague-Dawley (SD)	Charles River	94-CD-SIFE201
Rat: adult (4–15 months) female Hsd:RH-Foxn1^rnu^	Envigo	005

**Other**		

μ-Plate 24 Well Black ID 14 mm	Ibidi	82426
10x objective	Leica	11506505
6-well culture plate Nunclon Delta	Thermo Fisher Scientific	140675
Aquarium air curtain	Aquarium filters	N/A
Butyl gloves for DCM	Thermo Fisher Scientific	11-394-8A
Carbogen (95% O_2_ / 5% CO_2_)	Strandmöllen	N/A
Cooling unit	Julabo	FL300
Cover glass forceps	Fine Science Tools	11073-10
Fume hood	Iab Lab	N/A
Gas diffuser	Aquarium filters	N/A
Glass Petri dish, 100 × 15 mm	Thermo Fisher Scientific	455742
Glass vials with lid (20 mL)	Sigma-Aldrich	DWK986546
Laminar hood Mars Class 2	LaboGene	N/A
Long-round tip spatula	Fine Science Tools	10090-17
Mayo-Noble scissors	Fine Science Tools	14014-17
Metal nut (M6)	RS PRO	189-591
Micro-filter-candle	Robu	18104
Millicell culture inserts	Millipore	PICM0RG50
Moria perforated spoon	Fine Science Tools	10370-18
Nitrile gloves	VWR	112-2371
Paintbrush n°2	Slöjd-Detaljer	0171-0000
Paintbrush n°6	Slöd-Detaljer	1421-6031
Parafilm	Bemis	PM992
Pasteur pipettes	VWR	612-1701
Petri dish, 100 × 15 mm	Thermo Fisher Scientific	150350
Razor blade (Gillette Silver Blue)	Amazon	N/A
Rodent guillotine	World Precision Instruments	DCAP-M
Round cover glass (OD 13 mm)	Nordic Biolabs	111530
Rubber teats, silicone	VWR	BIBBBPP002
Shaking platform (VXR basic Vibrax)	IKA	0002819000
Straight bone cutter	Fine Science Tools	16152-11
Straight forceps	Fine Science Tools	11000-1
Straight sharp-blunt scissors	Fine Science Tools	14008-14
Super Glue	Loctite	2062278
Syringe filter unit (0.2 μm)	Sarstedt	83.1826.001
Syringe filter unit (0.45 μm)	Sarstedt	83.1826
Syringe (60 mL)	Thermo Fisher Scientific	14-955-455
Tubing system	Ley Rubber	FB50857
TCS SP8 point laser scanning confocal microscope	Leica	N/A
Vibratome	Leica	Leica VT1200 S
Whatman round filter paper	Fisher Scientific	10292221

**Software and algorithms**		

Vision 4D	Arivis	www.arivis.com

## Materials and equipment

**Table:** MgSO_4_·7H_2_O stock (2 M) solution

Reagent	Final concentration	Amount
MgSO_4_**·**7H_2_O	2 M	49.3 *g*
ddH_2_O	N/A	∼ 100 mL
**Total**	**N/A**	**100 mL**

Dissolve using a magnetic stirrer. Store at 4°C for up to six months.

**Table:** CaCl_2_·2H_2_O stock (2 M) solution

Reagent	Final concentration	Amount
CaCl_2_**·**2H_2_O	2 M	29.4 *g*
ddH_2_O	N/A	∼ 100 mL
**Total**	**N/A**	**100 mL**

Dissolve using a magnetic stirrer. Store at 4°C for up to six months.

**Table:** MgCl_2_·6H_2_O stock (2 M) solution

Reagent	Final concentration	Amount
MgCl_2_·6H_2_O	2 M	40.6 *g*
ddH_2_O	N/A	∼ 100 mL
**Total**	**N/A**	**100 mL**

Dissolve using a magnetic stirrer. Store at 4°C for up to six months.

**CRITICAL:** As calcium chloride is hazardous (see safety information and chemical handling). Wear eye protection/face protection.

**Table:** N-methyl-D-glucamine (NMDG)- 4-(2-hydroxyethyl)-1-piperazineethanesulfonic acid (HEPES) artificial cerebrospinal fluid (aCSF)

Reagent	Final concentration	Amount (1X)
NMDG	92 mM	17.96 *g*
HCl (37%)	∼ 0.26%	∼ 7 mL
KCl	2.5 mM	0.19 *g*
NaH_2_PO_4_	1.25 mM	0.17 *g*
NaHCO_3_	30 mM	2.52 *g*
HEPES	20 mM	4.77 *g*
Glucose	25 mM	4.51 *g*
Sodium ascorbate	5 mM	0.99 *g*
Thiourea	2 mM	0.15 *g*
Sodium pyruvate	3 mM	0.33 *g*
MgSO_4_**·**7H_2_O (2M Stock)	10 mM	5 mL
CaCl_2_**·**2H_2_O (2M Stock)	0.5 mM	0.25 mL
ddH_2_O	N/A	∼ 1000 mL
**Total**	**N/A**	**1 l**

NMDG-HEPES aCSF saturated with carbogen (95% O_2_ / 5% CO_2_) is used to maintain the brain tissue alive during the cutting procedure. This buffer solution contains a high concentration of HEPES that provides a strong pH buffering as well as antioxidant agents such as thiourea and ascorbic acid-lowering oxidative stress, which increase cell survival. NMDG-HEPES aCSF should be prepared a day before the experiment as described in the table below using sterile deionized water.

Store NMDG-HEPES aCSF at 4°C overnight.

∗ Titrate pH to 7.3–7.4 with 7 mL ± 0.2 mL of hydrochloric acid (37% HCl). pH adjustment *s*hould ideally be performed prior to adding CaCl_2_ and MgSO_4_ to avoid precipitation. Then, the osmolarity of the solution is measured and adjusted to 300–305 mOsm/kg by adding ddH_2_O if osmolarity is too high and 1 mg/mL glucose if osmolarity is too low. This preparation was adapted from.^14^

**CRITICAL:** Thiourea, HCl, sodium pyruvate, CaCl_2_, are hazardous (see safety information and chemical handling). Wear protective gloves, safety goggles, protective clothing, and a face mask should be mandatory.

**Table:** KPBS stock (0.12 M) solution

Reagent	Final concentration	Amount
KH_2_PO_4_	22.05 mM	6.86 *g*
K_2_HPO_4_	102.57 mM	40.8 *g*
NaCl	943.16 mM	126.0 *g*
ddH_2_O	N/A	∼ 2286 mL
**Total**	**0.12 M**	**2286 mL**

Dissolve using a magnetic stirrer and adjust pH to 7.0–7.4. Store at 4°C for up to six months.

**Table:** Sodium azide stock (10%) solution

Reagent	Final concentration	Amount
NaN_3_	10%	10 *g*
ddH_2_O	N/A	∼ 100 mL
**Total**	**N/A**	**100 mL**

Dissolve using a magnetic stirrer. Store at 4°C for up to twelve months.

**CRITICAL:** Sodium azide is hazardous (see safety information and chemical handling) and must be always handled under a fume hood and with protective measures.

**Table:** Triton X-100 stock (10%) solution

Reagent	Final concentration	Amount
Triton X-100	10%	10 mL
ddH_2_O	N/A	∼ 90 mL
**Total**	**N/A**	**100 mL**

Dissolve using a magnetic stirrer. Store at 4°C. Solution is stable for months.

**CRITICAL:** Triton X-100 is hazardous (see safety information and chemical handling) and needs to be always handled under a fume hood and with protective measures.

**Table:** Wash buffer for brain slice cultures

Reagent	Final concentration	Amount
HBSS (no calcium, no magnesium, no phenol red) (1X)	N/A	∼ 50 mL
CaCl_2_**·**2H_2_O (2M)	1.26 mM	31.5 μL
MgCl_2_·6H_2_O (2M)	0.49 mM	12.3 μL
MgSO_4_**·**7H_2_O (2M)	0.41 mM	10.3 μL
HEPES	19.97 mM	238 mg
D-(+)-Glucose	11.13 mM	100.3 mg
Antibiotic-Antimycotic (100X)	2.5X	1.25 mL
Penicillin-Streptomycin (10,000 U/mL) (100X)	2.5X	1.25 mL
**Total**	**N/A**	**50 mL**

**CRITICAL:** The solution must be prepared on the day of the experiment and saturated with carbogen (95% O_2_ / 5% CO_2_) for at least 10 min and stored at 4°C.

**Table:** Brain slice culture medium

Reagent	Final concentration	Amount
BrainPhys (1X)	N/A	48 mL
B-27 Supplement (minus vitamin A) (50X)	1X	1 mL
L-Glutamine 200mM (100X)	2 mM (1X)	0.5 mL
Antibiotic-Antimycotic (100X)	1X	0.5 mL
**Total**	**N/A**	**50 mL**

**CRITICAL:** The solution must be freshly prepared, sterilized by filtration through a 0.2 μm syringe filter unit and equilibrated to 5% CO_2_ at 37°C for at least 30 min before use. This solution is suitable for storage at 4°C and can used within two weeks if L-Glutamine 200 mM is freshly added every time before use.

***Alternatives:*** L-Glutamine 200 mM can be substituted with 1% GlutaMAX Supplement (100X) which is more stable and does not spontaneously degrade. This solution can be stored ad 4°C and used for one week.

**Table:** KPBS (0.02 M)

Reagent	Final concentration	Amount
KPBS stock (0.12 M)	0.02 M	100 mL
ddH_2_O	N/A	500 mL
**Total**	**0.02 M**	**600 mL**

Store at room temperature (around 20°C–23°C). Solution is stable for weeks.

**Table:** Bacteriostatic preservative

Reagent	Final concentration	Amount
Sodium azide (10%)	0.01%	100 μL
KPBS (0.02 M)	0.0198 M	9.9 mL
**Total**	**N/A**	**10 mL**

Prepare fresh.

***Note:*** This table describes the volume required for storing 10 brain sections, considering 1 mL per section.

**Table:** Blocking solution

Reagent	Final concentration	Amount
KPBS (0.02 M)	0.0182 M	27.3 mL
Triton X-100 (10%)	0.3%	900 μL
Donkey serum	5%	1.5 mL
Sodium azide (10%)	0.01%	300 μL
**Total**	**N/A**	**30 mL**

Prepare fresh.

***Note:*** This table describes the volume required for 10 brain sections, considering 1 mL per section at each incubation step (permeabilization, primary antibody, secondary antibody conjugated to fluorescent-dyes).

***Alternatives:*** Use serum specific to the host animal that the secondary antibody was generated in (i.e., goat serum for goat anti-rabbit secondary antibodies). Note that the serum cannot be from the same host species of the primary antibody (i.e., goat serum cannot be used for goat primary antibodies).

**Table:** Methanol series (20%–80%)

Reagent	Final concentration	Amount
Methanol (100%)	20%	20 mL
ddH_2_O	80%	80 mL
**Total**	**20%**	**100 mL**

**Table:** 

Reagent	Final concentration	Amount
Methanol (100%)	40%	40 mL
ddH_2_O	60%	60 mL
**Total**	**40%**	**100 mL**

**Table:** 

Reagent	Final concentration	Amount
Methanol (100%)	60%	60 mL
ddH_2_O	40%	40 mL
**Total**	**60%**	**100 mL**

**Table:** 

Reagent	Final concentration	Amount
Methanol (100%)	80%	80 mL
ddH_2_O	20%	20 mL
**Total**	**80%**	**100 mL**

Store at room temperature (around 20°C–23°C) for up to one week.

**CRITICAL:** Methanol is hazardous (see safety information and chemical handling) and needs to be always handled under a fume hood and with protective measures.

**Table:** Dichloromethane (DCM)/methanol mixture solution

Reagent	Final concentration	Amount
Methanol (100%)	33.33%	3.4 mL
DCM	66.67%	6.6 mL
**Total**	**N/A**	**10 mL**

Prepare fresh.

**CRITICAL:** DCM is hazardous (see safety information and chemical handling) and needs to be always handled under a fume hood and with protective measures.

***Note:*** This table describes the volume required for 10 brain sections, considering 1 mL per section.

### Safety information and chemical handling

**Table:** 

Chemical, IUPAC name	Chemical safety information	Health hazard information	Handling precautions
Paraformaldehyde, Polyoxymethylene	Flammable Health hazard Corrosive Irritant	Harmful if swallowed or inhaled. Causes skin irritation. May cause allergic skin reaction. Causes serious eye damage. May cause respiratory irritation. Suspected carcinogen.	Wash hands thoroughly after handling. Work in fume hood. Wear protective gloves. Wear safety goggles. Wear protective clothing.
Sodium Azide	Acute toxic Health hazard Environmental hazard	Highly toxic through skin contact, inhalation, and ingestion. Acute CNS and cardiovascular effects. Irritation to eyes, skin, and respiratory tract. Chronic exposure may result in liver, spleen, and kidney damage. Very toxic to aquatic life.	Wash hands thoroughly after handling. Work in fume hood. Wear protective gloves. Wear safety goggles. Wear protective clothing.
Triton X-100, polyethylene glycol tert-octylphenyl ether	Irritant Environmental hazard	Harmful if swallowed. Causes serious eye irritation. Toxic for aquatic life with long term consequences.	Wash hands thoroughly after handling. Wear safety goggles. Wear protective clothing.
Methanol	Flammable Health hazard Acute toxic	Exposure to excessive vapor causes eye irritation, headache, fatigue, and drowsiness. High concentrations can produce CNS depression and optic nerve damage. Can be absorbed through skin. Swallowing may cause death.	Wash hands thoroughly after handling. Work in fume hood. Wear protective gloves. Wear safety goggles. Wear protective clothing.
Dicholoromethane (DCM), dicholoromethane	Health hazard	The acute effects of methylene chloride inhalation consist mainly of decreased visual, auditory, and motor functions. DCM is highly volatile and can be absorbed through the skin. It can cause tissue necrosis. Suspected carcinogen.	Wash hands thoroughly after handling. Work in fume hood. Wear double gloves. Double gloves involve wearing butyl gloves over nitrile gloves. Wear safety goggles. Wear protective clothing.
Isoflurane, 2-chloro-2-(difluoromethoxy)-1,1,1-trifluoro-ethane	Health hazard	May cause eye, skin, or respiratory system irritation. May cause damage to cardiovascular, Central Nervous System through prolonged or repeated exposure. May cause drowsiness or dizziness. Toxicological properties have not been thoroughly investigated.	Work in fume hood. Avoid prolonged or repeated exposure Keep the container tightly closed. Store in accordance with information listed on the product insert.
Thiourea, Thiourea	Health hazard Environmental hazard	Harmful if swallowed. Suspected of causing cancer. Suspected of damaging fertility. Suspected of damaging the unborn child. Toxic to aquatic life with long lasting effects.	Wash skin thoroughly after handling. Do not eat, drink or smoke when using this product. Avoid release to the environment.
Hydrochloric acid, Chlorane	Acute toxic Corrosive	May be corrosive to metals. Causes severe skin burns and eye damage. May cause respiratory irritation.	Work in fume hood. Keep only in original packaging.
Sodium pyruvate, Sodium 2-oxopropanoate	Health hazard	May cause an allergic skin reaction. Causes serious eye irritation.	Wash skin thoroughly after handling.
Calcium chloride, calcium; dichloride	Health hazard	Causes serious eye irritation.	Wash skin thoroughly after handling.
Loctite® Liquid Super Glue	Health hazard	Bonds skin in seconds. Combustible liquid. Causes skin irritation. Causes serious eye irritation. May cause respiratory irritation.	Store in a well-ventilated place. Keep container tightly closed. Store in a well-ventilated place. Keep cool. Store locked up.

## Step-by-step method details

### Acute brain slices preparation

**Timing: 1 h**

As this protocol is designed to prepare acute brain slices for culturing, it is important to autoclave all surgical instruments and materials before use. Wearing protective gloves, safety goggles, protective clothing, and a face mask is required.

1. Setting up the cutting station.
  a. To reduce contamination, use water and then 70% ethanol to clean all surfaces including benchtop, vibratome, buffer tray, and tubing system used for diffusing the carbogen. Submerge the razor blade in 70% ethanol and let it dry for 5 min.
  b. Vibratome set up.
    i. Insert the razor blade into the blade holder and turn the vibratome on.
    ii. Check the blade’s position using the VibroCheck (see Leica VT1200 S user manual, pages 34–39).
    iii. Install the double-walled buffer tray (Leica VT1200 S user manual, pages 34–35) and use a silicone tube to connect a gas diffuser to the cylinder of compressed carbogen. The gas diffuser can be either a small micro-filter-candle or an aquarium air curtain.
    iv. Turn on the cooling unit connected to the buffer tray and set it up to 3°C at least 10 min before starting the preparation.
  c. Fill the buffer tray with 300 mL of pre-chilled NMDG-HEPES aCSF solution and maintain it with low carbogen bubbling to maintain minimal turbulence inside the buffer tray during the cutting.
  d. Place the vibratome’s specimen plate, a straight forceps, a Moria perforated spoon, a glass petri dish, a single Whatman round filter paper, the liquid super glue, and one razor blade on the benchtop next to the vibratome (Figure 1A).

**Figure 1:**
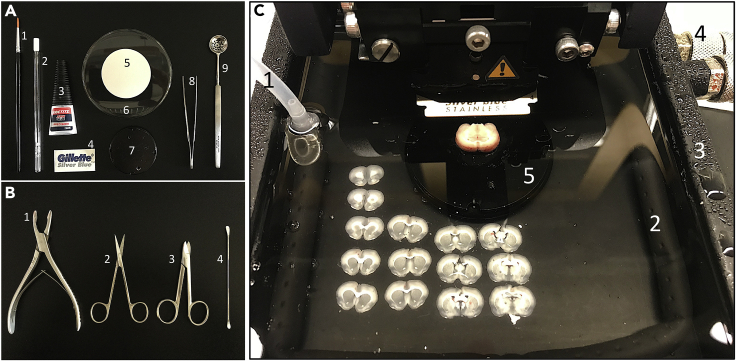
Brain slicing (A) Material for preparing the tissue block. Paintbrush n°2 (1), micro-filter candle (2), liquid super glue (3), razor blade (4), single Whatman round filter paper (5), glass petri dish (6), vibratome’s specimen plate (7), straight forceps (8), and Moria perforated spoon (9). (B) Surgical instruments for brain dissection. Straight bone cutter (1), straight sharp-blunt scissors (2), Mayo-Noble scissors (3), and long-round tip spatula (4). (C) Vibratome sectioning. Silicone tube (1) is connected to an aquarium air curtain (2) surrounding the buffer tray (3). Connection of the cooling unit to the buffer tray (4). Vibratome’s specimen plate submerged into the chilled NMDG-HEPES aCSF solution holding the brain tissue block (5).
2. Brain dissection and vibratome sectioning.
  a. Before starting the brain dissection.
    i. Prepare straight sharp-blunt scissors, Mayo-Noble scissors, straight bone cutter and long-round tip spatula (Figure 1B).
    ii. Add 80 mL of pre-chilled NMDG-HEPES aCSF solution into a 100 mL beaker, keep it inside a bucket with ice and continue carbogen bubbling using a small micro-filter-candle.
    iii. Prepare 50 mL of wash buffer and keep it in continuous carbogen bubbling using a micro-filter-candle.
  b. Surgery.
    i. Decapitation of the animal is performed with a rodent guillotine following depth anesthesia using an intraperitoneal injection with pentobarbital sodium (30–50 mg/kg).
    ii. Take the animal's head and remove the skin of the scalp using straight sharp-blunt scissors.
    iii. Using straight sharp-blunt scissors, make a 1 cm lateral/ventral incision cutting both sides of basioccipital bone through the foramen magnum at the occipital bone.
    iv. Insert the scissors again into the foramen magnum and cut dorsally alone the middle line of the interparietal, parietal, and frontal bones until it reaches the nasal bone. The cutting must be superficial and carefully performed to avoid damaging the underlying brain. Take Mayo-Noble scissors and cut the nasal bone transversally at the level of the premaxilla.
    v. Use the straight bone cutter to remove the nasal, frontal, parietal, and interparietal bones on both sides, starting from the rostral towards the caudal direction, to expose the brain.
    vi. Rinse the brain with 10 mL of pre-chilled NMDG-HEPES aCSF saturated with carbogen and then take a long-round tip spatula to gently scoop out the intact brain into the beaker prepared in step 2a.
  c. Brain slicing.
    i. Take the brain with a Moria perforated spoon and place it on a Whatman round filter paper in a 100 × 15 mm glass petri dish to prepare a tissue block. The tissue block can be prepared by cutting the brains at different angles depending on the region of interest. To prepare cortical coronal slices, the olfactory bulb and the cerebellum were removed, and the brain glued up vertically.
    ii. Lift the brain tissue block with straight forceps and fix it on the specimen plate with enough liquid super glue. Immediately, transfer the specimen plate into the buffer tray filled with chilled NMDG-HEPES aCSF continuously saturated with carbogen.
    iii. Use the vibratome’s control panel to set up the desired speed, amplitude of the vibration, and thickness of the slices which can be from 250 to 300 μm. The following parameters were used in this protocol: 0.14 mm/s speed, 1.10 mm amplitude and 275 μm slice thickness. The sectioning started from ventral towards dorsal part of the brain (Figure 1C).

**CRITICAL:** all animal procedures must follow the policies and guidelines established in your organization (university or company).

**CRITICAL:** Wear safety goggles to avoid accidents with the bone shards during the brain dissection.

**CRITICAL:** The tissue block preparation cannot take more than 30 sec; otherwise, it will affect the quality of the slices.

### Brain slice culturing

**Timing: 30**–**45 min**

After brain slicing, transfer the brain slices from the vibratome’s buffer tray to a 100 × 15 mm petri dish containing oxygenated pre-chilled wash buffer using a paintbrush n°6 and take them into a laminar flow hood.

**Figure 2:**
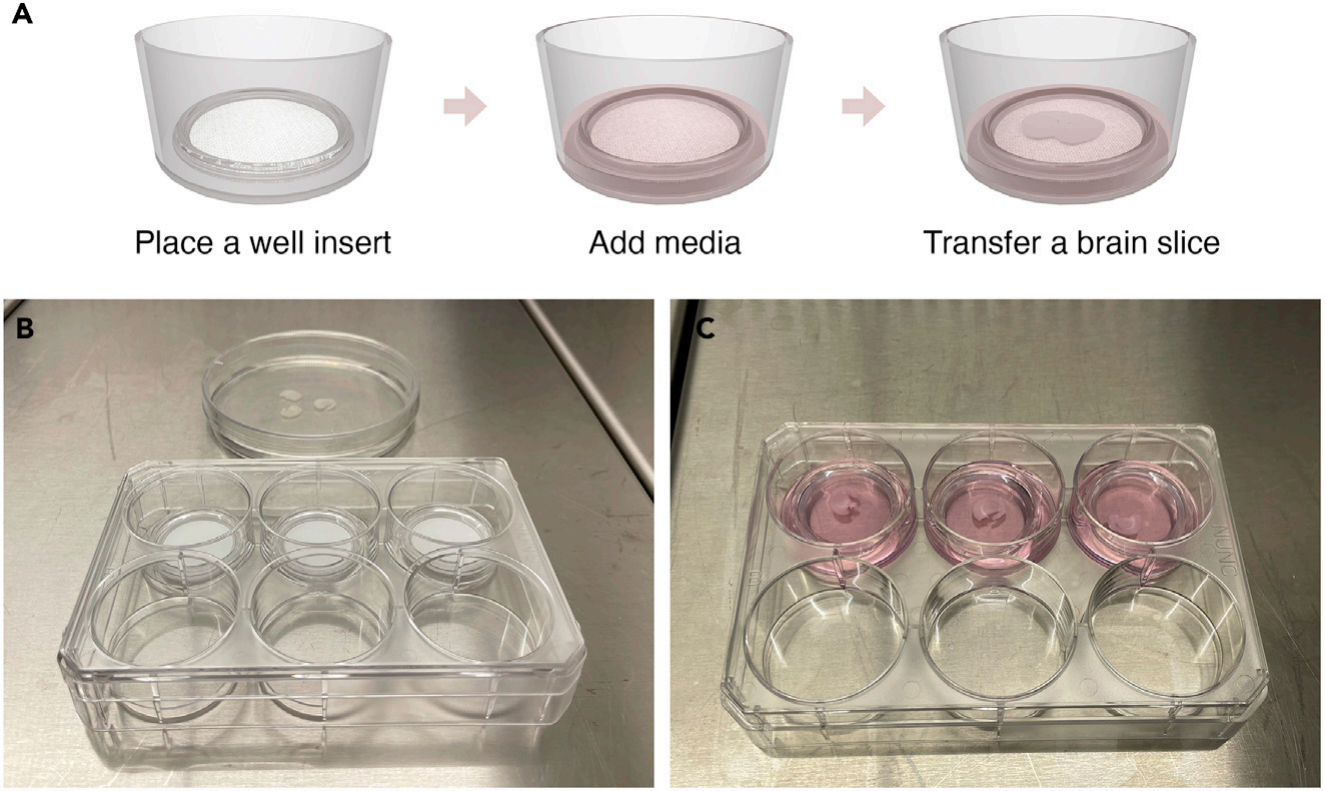
Brain slice culturing (A) Graphical illustration of the steps for starting brain slice culture. (B) Photograph of Millicell culture inserts placed in a 6-well culture plate. On the back, a petri dish containing 3 brain slices in wash buffer. (C) Photograph of brain slices positioned on top of Millicell culture insert and immersed in culture medium.
  c. Return the 6-well culture plate now containing the brain slices in culture medium to the 37°C incubator with 5% CO_2_.

3. Preparation of equipment and buffers for brain slice culture.
  a. Ensure that the following necessary equipment is organized for quick access: 6-well culture plate, Millicell culture inserts, autoclaved cover glass forceps and a paintbrush n°6 for transferring the brain slices.
  b. Clean the brush with 70% ethanol, let it dry for 5 min and rinse with deionized water thoroughly.
  c. Add 1 mL of brain slice culture medium to each well of a 6-well culture plate.
  d. Place a Millicell culture insert into each well using flat cover glass forceps and add an additional 0.5 mL of slice culture media on top of each insert.
  e. Place the 6-well culture plate in the incubator at 37°C with 5% CO_2_ for at least 30 min to ensure that the culture medium is pre-warmed before brain slices are plated.
4. Brain slice culture preparation.
  a. Place the petri dish containing the brain slices in wash buffer in the laminar flow hood and rinse the brain slices twice with fresh wash buffer equilibrated at physiological temperature (37°C).
  b. Manually transfer individual brain slices to each culture insert in 6-well culture plate using the paintbrush n°6. Perform this step carefully to avoid damaging the tissue (Figure 2, Methods video S1).

**CRITICAL:** Make sure that no areas of the brain slices are folded or wrinkled.

***Note:*** Brain slices submerged with media do not attach to the membrane of the Millicell culture insert and remain free-floating.

5. Brain slice culture maintenance.
  a. Every second day, remove ∼ 1 mL of culture medium from the bottom of each well and the excess medium that collects in the Millicell culture insert without disturbing the brain slice.
  b. Add 1 mL of fresh and pre-warmed culture medium to the bottom of each well and an additional 0.3 mL inside the Millicell culture insert to submerge the brain slice.
  c. Maintain brain slices in culture as long as required by the experimental plan.

**CRITICAL:** The culture medium needs to equilibrate at 37°C with 5% CO_2_ for 30 min before transferring the brain slices and before every medium change. Maintain the brain slices in culture under sterile conditions.

***Note:*** (1) Transfer an entire rat coronal brain slice per Millicell culture insert or 1–2 coronal brain slice-separated hemispheres depending on your unique experimental conditions. If more than 1 brain slice is plated, avoid slice overlap and ensure that slices do not contact the sides of the insert. (2) In our experience, brain slices from 275 μm thickness are not disturbed by using a paintbrush to transfer them. Alternatively, an inverted glass Pasteur pipette can be used to position individual slices in each Millicell culture insert although this will transfer wash buffer to the culture plate. Excess wash buffer can be removed by replacing the culture media with fresh and pre-warned culture media immediately after transferring. It is important to avoid prolonged exposure to the wash buffer in culture since it can affect brain slice health.

Methods video S1. Video showing how to use a paintbrush to transfer brain slices from petri dish to 6-well culture plate containing Millicell culture inserts, related to “Brain slice culturing” step 4b

### Immunostaining of brain slice cultures

**Timing: 5 days**

**Timing: 45 min (for step 6)**

**Timing: 16 h (for step 7)**

**Timing: 3–4 days (for step 8)**

6. Fixation of brain slices.
  a. Remove the culture medium and add 1 mL of 4% paraformaldehyde (PFA) solution pH 7.4 into the well and an additional 1 mL on top of the brain slice and fix for 20 min at room temperature (20°C–23°C).
  b. Gently transfer each fixed brain slice from the Millicell culture insert to a glass vial with lid containing 1 mL KPBS using a paintbrush. This will ensure compatibility with clearing reagents that will be used in the next steps.
  c. Wash the slice 2 × 5 min with KPBS at room temperature (20°C–23°C) by adding 1 mL of KPBS per vial.

***Note:*** Ensure that the volume of fixative is sufficient to fully immerse the brain slice.

**CRITICAL:** Do not fix brain slices longer than recommended. Immunostaining can otherwise be compromised due to excessive cross-linking of proteins. PFA is hazardous (see safety information and chemical handling) and needs to be always handled under a fume hood and with protective measures.

**Pause Point:** The fixed and washed brain slices can be stored at 4°C in bacteriostatic preservative (0.01% sodium azide in KPBS). Samples are stable for a few weeks.

7. Permeabilization and blocking of brain slices.
  a. Remove the KPBS.
  b. Permeabilize and block the brain slice by adding 1 mL of blocking solution in each vial.
  c. Incubate overnight at room temperature (20°C–23°C), or at 4°C for two days.

8. Antibody staining of brain slice.
  a. Prepare the primary antibody solution diluted (for this experiment rabbit anti-Iba1, 1:1000) in 1 mL of blocking solution.
  b. Remove the blocking solution from the glass vial containing the brain slice.
  c. Add 1 mL of primary antibody solution.
  d. Incubate for 48–72 h at room temperature (20°C–23°C).
  e. Remove the primary antibody solution.
  f. Wash the sample 3 × 20 min with KPBS at room temperature on a shaking platform at mild speed (100 RPM).
  g. Prepare the secondary antibody solution diluted 1:200 in 1 mL of blocking solution and add DAPI (1:1000) for nuclear stain.
    ***Note:*** Keep the secondary antibody solution protected from light. For example, by covering the tube and glass vial containing the antibody solution with aluminum foil.
  h. Remove the KPBS from the glass vial containing the brain slice.
  i. Add 1 mL of secondary antibody solution.
  j. Incubate for 24–48 h at room temperature (20°C–23°C).
  k. Remove the secondary antibody solution.
  l. Wash the sample 3 × 20 min with KPBS at room temperature on a shaking platform at mild speed (100 RPM).
    **Pause Point:** The immunostained and washed brain slices can be stored at 4°C in bacteriostatic preservative (0.01% sodium azide in KPBS) while protected from light for up to two weeks. However, we recommend continuing with clearing and imaging right after staining because the fluorescence decreases over time.
    **CRITICAL:** Throughout the entire staining procedure, be careful not to disrupt the brain slices when removing and adding solutions. Avoid drying out the brain slices. Perform each step on a mildly shaking platform (100 RPM) for homogeneous permeabilization and staining of the tissue. Seal the glass vial during incubation using a lid or parafilm to avoid excess evaporation.

### Clearing of immunolabelled brain slices

**Timing: 1 day**

After staining, the sample is ready to be cleared for imaging of the entire brain slice. The steps in this process are intended to remove water and lipids from the tissue and match the refractive index throughout the tissue.

9. Dehydrate brain slices in methanol.
  a. Prepare the methanol series (20%, 40%, 60%, 80%, 100%).
  b. Remove the KPBS from the glass vial containing the brain slice.
  c. Add 1 mL of 20% methanol and incubate 10 min at room temperature (20°C–23°C) on a shaking platform at mild speed (100 RPM).
  d. Remove the methanol solution and add 1 mL 40% methanol. Incubate for 10 min at room temperature (20°C–23°C) on a shaking platform at mild speed (100 RPM).
  e. Remove the methanol solution and add 1 mL 60% methanol. Incubate for 10 min at room temperature (20°C–23°C) on a shaking platform at mild speed (100 RPM).
  f. Remove the methanol solution and add 1 mL 80% methanol. Incubate for 10 min at room temperature (20°C–23°C) on a shaking platform at mild speed (100 RPM).
  g. Remove the methanol solution and add 1 mL 100% methanol. Incubate for 10 min at room temperature (20°C–23°C) on a shaking platform at mild speed (100 RPM).

**CRITICAL:** Methanol is hazardous (see safety information and chemical handling) and needs to be always handled under a fume hood and with protective measures. Close the glass vials with the lid before positioning them on the shaking platform outside the fume hood.

10. Removal of lipids using DCM treatment.
  a. Remove the methanol solution.
  b. Add 1 mL of DCM/methanol mixture solution. Incubate for 1 h at room temperature (20°C–23°C) on a shaking platform at mild speed (100 RPM).
  c. Remove the DCM/methanol mixture solution.
  d. Add 1 mL of DCM. Incubate for 10 min at room temperature (20°C–23°C) on a shaking platform at mild speed (100 RPM).
  e. Remove the DCM.
  f. Add 1 mL of DCM. Incubate for 10 min at room temperature (20°C–23°C) on a shaking platform at mild speed (100 RPM).

**CRITICAL:** DCM is hazardous (see safety information and chemical handling) and needs to be always handled under a fume hood and with protective measures. Close the glass vials with the lid before positioning them on the shaking platform outside the fume hood.

11. Clear the tissue using ethyl cinnamate.
  a. Add 500 μL of ethyl cinnamate per well in the black Ibidi μ-Plate 24 well plate with flat and clear bottom for high throughput microscopy.
  b. Remove the DCM from the glass vial containing the brain slice.
  c. Add 500 μL of ethyl cinnamate to avoid DCM carry-over.
  d. Gently transfer the cleared brain slice from the glass vial to the Ibidi μ-Plate 24 well plate containing ethyl cinnamate using a paintbrush.
  e. Incubate overnight at 4°C protected from light.

**Figure 3:**
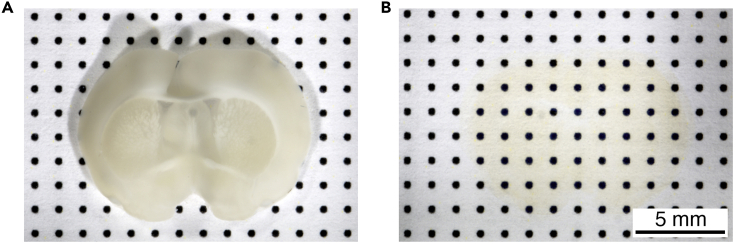
Brain slice clearing (A) Image of a brain slice in KPBS after immunostaining. (B) Image of an immunostained brain slice after dehydration and clearing in ethyl cinnamate. Tissue is almost fully transparent with faint colorization.

***Note:*** The tissue becomes transparent after adding ethyl cinnamate (Figures 3A and 3B).

***Note:*** The No.1.5 polymer coverslip bottom of the Ibidi μ-Plate 24 well plate has the highest optical quality and is resistant ethyl cinnamate treatment.

**Pause Point:** The cleared brain slices can be stored at 4°C in ethyl cinnamate for weeks. However, we recommend imaging the samples the day after clearing because the fluorescence decreases over time.

**CRITICAL:** Be very careful not to dry the sample when changing solutions or transferring the tissue.

### Confocal imaging of cleared brain slices

**Timing: 5**–**30 min per slice**

We used Leica TCS SP8 point laser scanning confocal microscope with a 10x objective, but the prepared samples can be imaged with any inverted laser scanning confocal microscope equipped with appropriate objectives and filter sets and the imaging parameters would vary depending on the performed immunostaining and experimental goals. Here we used anti-Iba1 primary antibody in combination with Alexa Fluor 647 secondary antibody to visualize microglia in striatal slices. Leica dynamic filter was used for imaging. Imaging the sample using air objective or immersion objective with RI different from ethyl cinnamate (RI = 1.56) results in refractive index mismatch. Geometric distortion along the z-axis caused by RI mismatch can be correct via the formula provided below.

12. Flatten the immunostained and cleared brain slice.
  a. Reduce the volume of ethyl cinnamate so it just covers the brain slice.
  b. Place a round glass coverslip in the well on top of the brain slice.
  c. Place 3–4 metal nuts on top of the coverslip amounting to 7–10 g of weight in total to mechanically flatten the brain slice.
13. Image the brain slice by taking a z-stack throughout the thickness of the sample.
  a. Objective: 10x NA 0.3.
  b. Z-step 10 μm.
  c. Resolution: 1024 × 1024 px (pixel size = 1.52 μm).
  d. Scan speed: 400 MHz.
14. Correct for RI mismatch using the following formula.
  a. $Z_{corrected}=Z_{uncorrected}\frac{1.56}{{RI}_{objective\;immersion}}$

***Note:*** Z(corrected) is the actual height while Z(uncorrected) is the perceived height.

**Figure 4:**
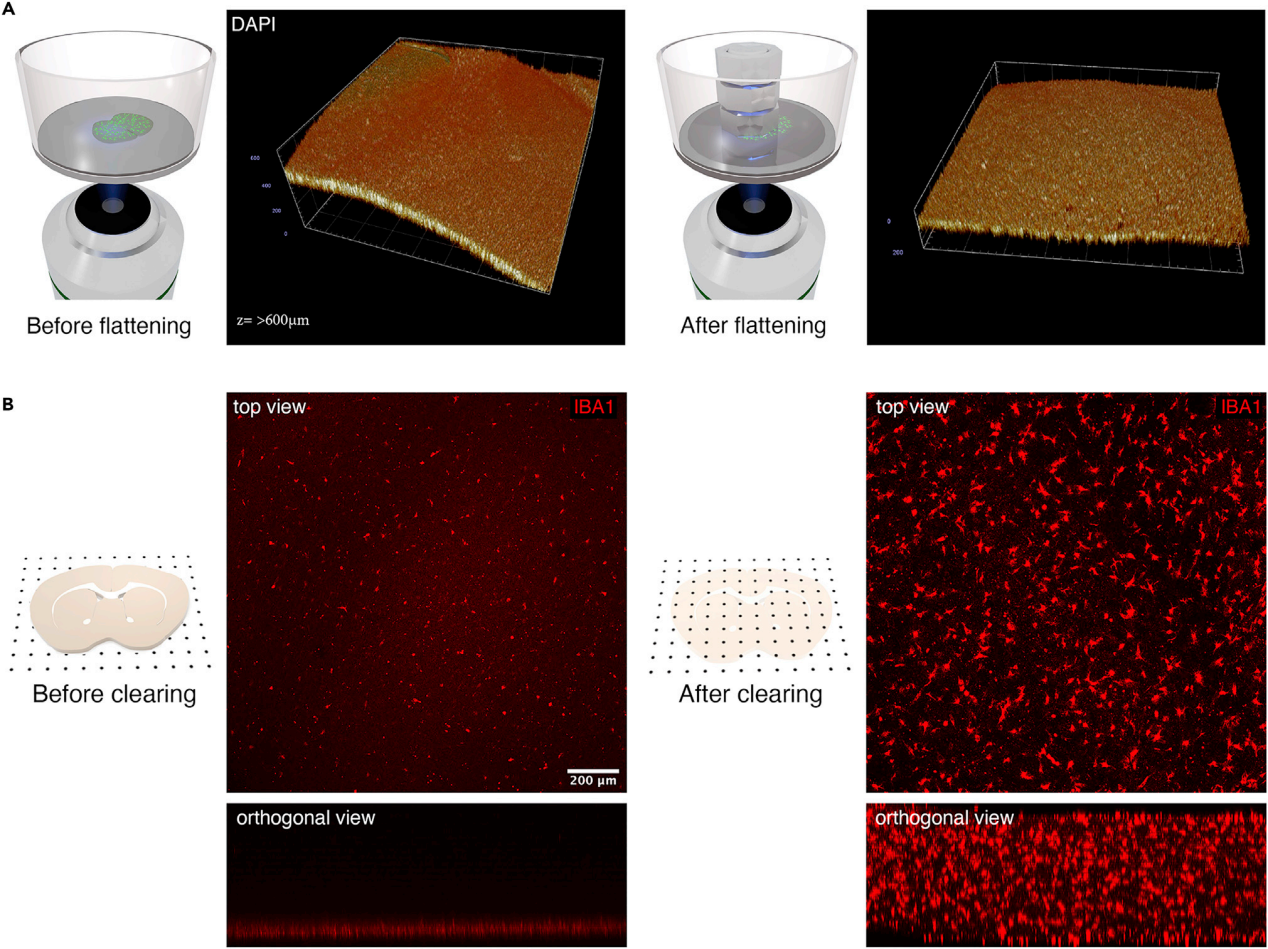
Flattening of cleared brain slices (A) 3D reconstruction of an image stack displaying a section of a cleared brain slice before (left) and after flattening (right). Flattening is an important step because distortions in brain slice geometry that occur during cutting and clearing (due to variable shrinkage rates of different brain tissue areas) lead to difficulties and limitations during image acquisition. (B) Images show an Iba1-immunostained microglial cells within the brain slice before (left) and after (right) clearing procedure was performed. Orthogonal view demonstrates how much information is lost in non-cleared tissue. Clearing allows imaging of the entire thickness of the brain slice. Methods video S1: Video showing how to use a paintbrush to transfer brain slices from petri dish to 6-well culture plate containing Millicell culture inserts. Related to “Brain slice culturing” step 4b.

15. Render 3D view in Vision 4D or similar software (Figures 4A and 4B).

**CRITICAL:** Metal nuts might reflect light and therefore impede imaging if a spinning disk confocal or a widefield microscopes are used. In this case, remove the nuts before imaging. In our experience, brain slices remain flattened if imaged right after the removal of the weight.

***Note:*** Care should be taken in handling the plates as the sample could move in the wells. We have not experienced brain slices moving during imaging.

## Expected outcomes

The first part of the protocol provides detailed instructions on how to prepare 275 μm thick adult rat brain slices (Figure 1) and place them in organotypic culture (Figure 2, Methods video S1). Proceeding with organotypic culture is optional as brain slices can be used for electrophysiological measurements immediately after cutting and fixed once the measurements are executed. The second part of the protocol provides detailed instructions and troubleshooting guidance for fluorescence immunostaining, clearing (Figure 3), flattening and confocal imaging of fixed brain slices (Figure 4). Importantly, the protocol takes advantage of widely available reagents and tools. Furthermore, ethyl cinnamate is used for refractive index-matching which is non-toxic and therefore allows imaging on multi-user microscopes without the need for an exhaust system. Therefore, we believe that the protocol can be easily adapted by other research groups and readily integrated into experimental plans.

## Limitations

Sample dehydration using methanol significantly reduces the intensity of endogenous fluorescence in 488 nm band. If retention of endogenous fluorescence is of crucial importance, 1-propanol adjusted to alkaline pH should be used for dehydration instead.^9^ Quantitative morphometric analysis of cleared brain slices might be affected by distortions caused by dehydrated tissue shrinkage. Furthermore, refractive index mismatch caused by using air objectives to image samples in ethyl cinnamate leads to geometrical distortion of the final image. Due to this effect, the sample will appear shortened along the z-axis and needs to be corrected for.

## Troubleshooting

### Problem 1

Low brain slice quality is a very common problem in electrophysiology and *ex vivo* cultures (associated with sections: “Acute brain slice preparation” steps n° 1–2 and “Brain slice culturing” steps n° 3–5). It can be due to several factors including osmolarity and pH of the buffer solution, oxygen deprivation, mechanical stress on the slices, and technical skills.

### Potential solution

Osmolarity and pH: be sure that all devices are properly calibrated before use.

Oxygen deprivation: this occurs during brain dissection and tissue block preparation; therefore, those steps should be carried out as quick as possible. It can be resolved by improving technical skills.

Mechanical stress: lift the slices carefully with the paintbrush and avoid disturbing the slices excessively while performing media changes.

### Problem 2

Strong staining only at tissue edges or antibody does not penetrate deeply into the tissue (associated with section: “Immunostaining of brain slice cultures” steps n° 6–8). This problem may be attributed to the permeabilization step or antibody incubation time.

### Potential solution

Increase Triton X-100 concentration (e.g., to 0.5%) and/or prolong antibody incubation steps (e.g., to 96 h, refreshing the solution after 48 h).

### Problem 3

High background signal and/or bright dots of staining in the tissue (associated with section: “Immunostaining of brain slice cultures” steps n° 6–8). This problem may be attributed to the precipitates present in PFA solution, insufficient blocking of the tissue, inadequate washing or improper (excessive) antibody concentration and/or incubation. The problem could also be contributed to residual presence of erythrocytes in microcirculation that can produce autofluorescence. If this is the case, it is recommended to perform saline perfusion during preparation of acute brain slices in order to clear the blood from the brain.

### Potential solution

It is recommended to filter the PFA solution through a 0.45 μm syringe filter unit to remove any particulate matter before fixation of the brain slices. Extend the time of blocking solution incubation and/or increase the frequency of washing steps after each antibody incubation step. Titer the antibody concentration upon first use. Centrifuge the secondary antibody stock.

### Problem 4

Extremely bright staining of the tissue sample (associated with section: “Clearing of immunolabelled brain slices” steps n° 9–11). This problem may be attributed to excessive drying/shrinkage of the sample when changing solutions or transferring the tissue during clearing.

### Potential solution

Leave a small amount of liquid left in the vial and apply the new solution immediately after removal.

### Problem 5

Tissue samples become opaque during long term storage (associated with section “Clearing of immunolabelled brain slices” steps n° 9–11). This issue might occur if there are aqueous solution (e.g., KPBS) in other wells in the same plate as the cleared tissue or the stored sample is exposed to increased humidity.

### Potential solution

Make sure that the plate with cleared samples only contains ethyl cinnamate. Seal the plate with parafilm.

## Resource availability

### Lead contact

Further information and requests for resources and reagents should be directed to and will be fulfilled by the lead contact, Janko Kajtez (janko.kajtez@med.lu.se).

### Materials availability

This study did not generate new unique materials. All materials are purchasable.

## Data Availability

All data needed to evaluate the conclusions in the paper are present in the paper. Additional data and codes related to this paper may be requested from the authors.
